# A Quantitative Assessment of Factors Affecting the Technological Development and Adoption of Companion Diagnostics

**DOI:** 10.3389/fgene.2015.00357

**Published:** 2016-01-28

**Authors:** Dee Luo, James A. Smith, Nick A. Meadows, A. Schuh, Katie E. Manescu, Kim Bure, Benjamin Davies, Rob Horne, Mike Kope, David L. DiGiusto, David A. Brindley

**Affiliations:** ^1^Department of Biological Basis of Behavior, University of PennsylvaniaPhildephila, PA, USA; ^2^The Oxford – UCL Centre for the Advancement of Sustainable Medical Innovation, University of OxfordOxford, UK; ^3^Nuffield Department of Orthopaedics, Rheumatology and Musculoskeletal Sciences, University of OxfordOxford, UK; ^4^Kinapse, WimbledonLondon, UK; ^5^National Institute of Health Research, Biomedical Research Centre, Molecular Diagnostic Centre, Oxford University HospitalsOxford, UK; ^6^Department of Biochemical Engineering, University College LondonLondon, UK; ^7^Botnar Research Centre, Nuffield Department of Orthopaedics, Rheumatology and Musculoskeletal Sciences, University of OxfordOxford, UK; ^8^Sartorius StedimGöttingen, Germany; ^9^The UCL School of Pharmacy, University College LondonLondon, UK; ^10^SENS Research FoundationMountain View, CA, USA; ^11^Stem Cell and Cellular Therapeutics Operations at Stanford University Hospital and ClinicStanford, CA, USA; ^12^USCF-Stanford Center of Excellence in Regulatory Science and InnovationSan Francisco, CA, USA; ^13^Centre for Behavioural Medicine, UCL School of Pharmacy, University College LondonLondon, UK; ^14^Harvard Stem Cell InstituteCambridge, MA, USA

**Keywords:** companion diagnostic, combinational therapy, risk:benefit appraisal, healthcare risk management, personalized medicine, stratified medicine, healthcare translation, clinical adoption

## Abstract

Rapid innovation in (epi)genetics and biomarker sciences is driving a new drug development and product development pathway, with the personalized medicine era dominated by biologic therapeutics and companion diagnostics. Companion diagnostics (CDx) are tests and assays that detect biomarkers and specific mutations to elucidate disease pathways, stratify patient populations, and target drug therapies. CDx can substantially influence the development and regulatory approval for certain high-risk biologics. However, despite the increasingly important role of companion diagnostics in the realization of personalized medicine, in the USA, there are only 23 Food and Drug Administration (FDA) approved companion diagnostics on the market for 11 unique indications. Personalized medicines have great potential, yet their use is currently constrained. A major factor for this may lie in the increased complexity of the companion diagnostic and corresponding therapeutic development and adoption pathways. Understanding the market dynamics of companion diagnostic/therapeutic (CDx/Rx) pairs is important to further development and adoption of personalized medicine. Therefore, data collected on a variety of factors may highlight incentives or disincentives driving the development of companion diagnostics. Statistical analysis for 36 hypotheses resulted in two significant relationships and 34 non-significant relationships. The sensitivity of the companion diagnostic was the only factor that significantly correlated with the price of the companion diagnostic. This result indicates that while there is regulatory pressure for the diagnostic and pharmaceutical industry to collaborate and co-develop companion diagnostics for the approval of personalized therapeutics, there seems to be a lack of parallel economic collaboration to incentivize development of companion diagnostics.

## Introduction

Cures for age-related diseases such as cardiovascular disease, diabetes mellitus, Alzheimer’s disease, and cancer, are increasingly unlikely to come from historic pharmaceutical industry models of therapeutic innovation and development. While the standard model of drug R&D utilized by pharmaceuticals has produced effective treatments to manage such diseases as hypertension, asthma, and arthritis, highly variable and increasingly more complex diseases such as cancer will require much more personalized approaches to patient treatment. The future of medicine, fueled by growth in complex biological data, lack of innovation in current pipelines, and political pressure, has therefore been shifting toward personalized medicine (La Thangue and Kerr, 2011). Personalized medicine, as defined by the National Academies Press, is “the ability to classify individuals into subpopulations that differ in their susceptibility to a particular disease or their response to a specific treatment. Preventive or therapeutic interventions can then be concentrated on those who will benefit, sparing expense and side effects for those who will not” (La Thangue and Kerr, 2011). Specifically in personalized medicine, there has been an increasing focus on the barriers to widespread adoption (Brindley et al., 2015). The current literature highlights cost-effectiveness, efficacy, reimbursement, and regulation (Davies et al., 2014; French et al., 2014) as the key concerns for adoption of current personalized medicines, but an aspect of personalized medicine that has recently come into the spotlight as an inseparable aspect of precision medicine is the role of companion diagnostics.

Companion diagnostics (CDx) are a critical component in advancing personalized medicine. Companion diagnostics were officially defined in 2014 by the Food and Drug Administration (FDA; Silver Springs, MD, USA) as “a medical device, often an *in vitro* device, which provides information that is essential for the safe and effective use of a corresponding drug or biological product” (Center for Devices and Radiological Health, 2015b). Biomarker based stratification assays designed to identify responsive patient subpopulations and detect patients more likely to experience adverse effects are becoming increasingly important to securing market share for high efficacy, high cost therapeutics (Amir-Aslani and Mangematin, 2010; Yip et al., 2015).

From a pharmaceutical development viewpoint, CDxs can contribute to the successful approval and market launch of high-risk but high benefit therapeutics. The success of Herceptin (Trastuzumab; Roche, Basel, Switzerland), a monoclonal antibody (mAb) that blocks the growth of malignant tumor cells, relies on the ability of a CDx to accurately identify the subpopulation (22%) of patients with HER2-positive early breast cancer (Trusheim et al., 2011)^.^ Failure to stratify the patient population will result in both costly interventions and unnecessary exposure to increased cardiotoxicities for patients who cannot benefit from the drug.

Herceptin was groundbreaking as the first targeted therapy to address the underlying causes of a predominantly age-related disease, and its CDx, HercepTest (Dako, Glostrup, Denmark), was the first CDx to enter clinical practice in 1998 [“Genentech: Herceptin^®^, (trastuzumab) Development Timeline, n.d.”]. However, few companion diagnostics/treatment (CDx/Rx) pairs have been approved for the market since Herceptin’s breakthrough (Jakka and Rossbach, 2013; Davies et al., 2014). There have been a limited total of 23 approved CDx for 11 personalized therapies and seven primary indications in the past 17 years (Center for Devices and Radiological Health, 2015a). The rates of technological development and clinical adoption of CDxs has been arguably limited relative to the extent of public and private investment.

Limited progress could be due to a number of reasons. There are numerous scientific challenges to the discovery and production of companion diagnostics and corresponding therapeutics (De Lecea and Rossbach, 2012). Equally important to the advancement of personalized therapies, however, are significant regulatory and economic challenges (Davis et al., 2009; Djalalov et al., 2011; Jakka and Rossbach, 2013).

Regulation for CDx and Rx is intrinsically connected, and this relationship may or may not be reflected in the economic incentives and disincentives driving innovation and development of CDx/Rx pairs. The FDA recently released the “*In Vitro* Companion Diagnostic Devices: Guidance for Industry and Food and Drug Administration Staff” on July 31st, 2014, suggesting that companion diagnostics and corresponding therapeutics be co-developed and evaluated concurrently (Guidance for Industry and Food and Drug Administration Staff, 2014).

**FIGURE 1:**
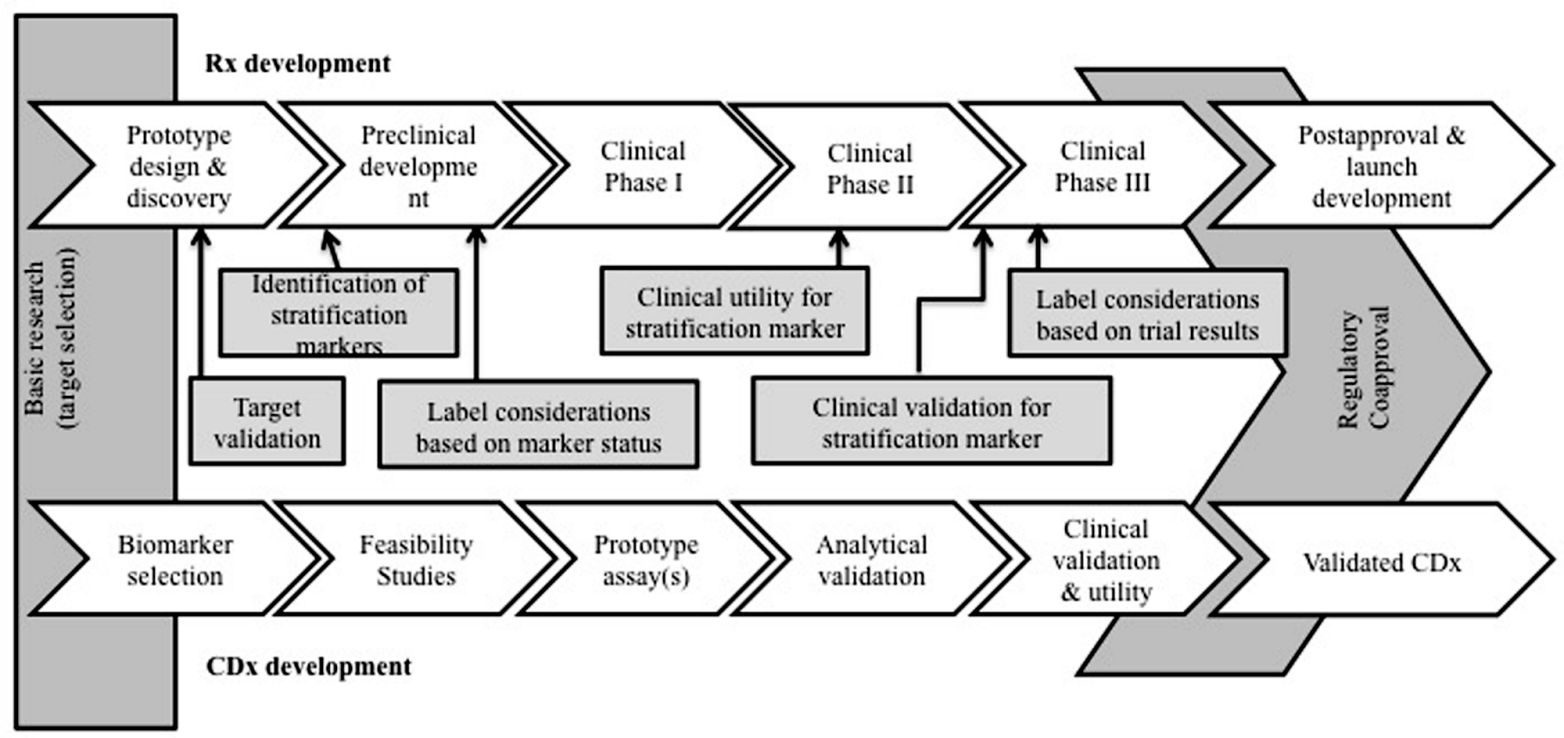
**Companion diagnostic development and regulation concomitant with drug therapy development and regulation.** The relationship between a companion diagnostic and therapeutic is unique and economic effects of co-development have yet to be fully realized.

This suggested regulatory link between companion diagnostics and their corresponding drugs can influence economic factors and affect the rate of market approval and adoption (Hinman et al., 2008; Yip et al., 2015). Specifically, the collaboration between two typically independent industries potentially presents challenges in aligning incentives between diagnostic developers and pharmaceutical manufacturers (Chiang and Million, 2011; Jørgensen, 2013). **Figure 1** shows the regulatory framework that underpins co-development. To date, the regulatory landscape for market approval and price determinations of CDx/Rx pairs is relatively unexamined and lacking quantitative analysis necessary for changes and improvements.

Therefore, it is important to analyze current, publically available data for companion diagnostics and corresponding therapeutics to identify drivers, limitations, and areas for more research in the current landscape. A systematic analysis and numerical data will identify factors affecting the CDx/Rx pair market and provide more information to assess the impact of future regulatory and policy changes in facilitating or limiting the development of companion diagnostics. This paper provides a data driven analysis of the CDx/Rx market by statistically analyzing and discussing factors that may incentivize, restrict, or create uncertainty in the development and adoption of companion diagnostics.

## Materials and Methods

### Inclusion Criteria

**FIGURE 2:**
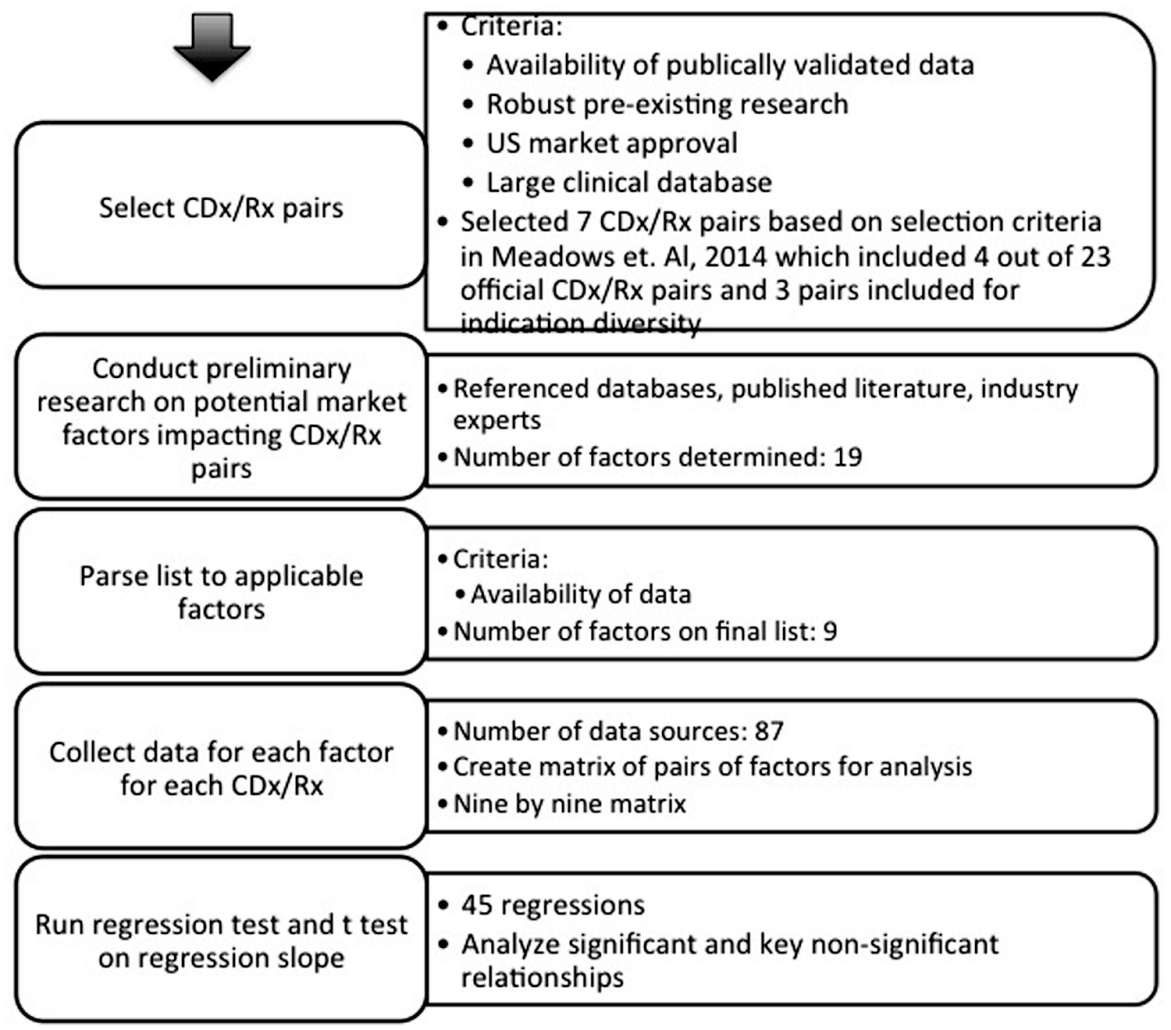
**Graphical representation of methodology.** Analysis proceeded in a stepwise fashion.

A systematic analysis was conducted with methodology set in **Figure 2**. Seven sets of personalized therapies were selected for analysis based on a series of case studies of personalized medicines and their CDx published in Meadows et al. (2015). These cases describe a number of indications over a period of 15 years; although not a comprehensive list, they represent a number of leading examples of successful stratification of therapeutics. While there are 23 FDA approved CDx for 11 personalized therapeutics, the availability of established, validated, and publically accessible data, along with the existence of robust prior research, limits the number of CDx/Rx pair available for examination to the seven personalized therapeutics presented in previous literature (Meadows et al., 2015). The majority of CDx/Rx pairs presented were in the field of oncology, such as Herceptin, Xalkori, Zelboraf (vemurafenib; Roche, Basel, Switzerland), and Vectibix (Panitumumab; Amben, CA, USA). Oncology contributes a large proportion of CDx/Rx due to its inherently highly stratified populations and extensive investment in biomarker discovery, but the development of companion diagnostics is not limited to oncological indications. Therefore, final list of CDx/Rx pairs attempts to capture multiple primary indications, including CDx/Rx pairs beyond those on the “List of FDA Approved Companion Diagnostics” (Center for Devices and Radiological Health, 2015a) to include the three highly personalized therapeutics and unofficial companion diagnostics examined in Meadows et al. (2015): Ziagen (abacavir sulfate; Viiv Healthcare, Brentford, UK), Selzentry (Maraviroc; Viiv Healthcare, Brentford, UK), and Kalydeco (Vertex, Cambridge, MA, USA). All three additional CDx/Rx pairs address indications (cystic fibrosis and HIV) in which specific mutations and tropisms are known at time of diagnosis and extensive prior literature is available to support their classification as personalized therapeutics (Meadows et al., 2015).

**Table 1** shows the finalized table of companion and pseudo-companion diagnostic and therapeutic pairs that meet the requirements of a large clinical database, pre-existing research, long duration on market, robust data, and US market approval.

**Table 1:** Select list of FDA approved personalized therapeutic/CDx pairs.

Primary indication	Drug (pharma)	CDx/approved year (diagnostic company)	Assay
Breast cancer	Herceptin (Roche, Basel, Switzerland)	HercepTest/1998 (Dako, Glostrup, Denmark)	SemiqIHC
		SPOT-light HER-2 CISH Kit/2008 (Life Technologies, South San Francisco, CA, USA)	CISH
		Bond Oracle Her2 INC System/2012 (Leica Biosystem, Nussloch, Germany)	SemiqIHC
		PATHVYSION HER-2 DNA Probe Kit/2013 (Abbott, IL, USA)	FISH DNA Probe
		HER2 FISH PharmDx Kit/2005 (Dako)	qFISH
		INFORM HER2 Dual ISH/2011 (Roche)	CISH

Melanoma	Zelboraf (Roche)	COB AS 4800 BRAF V600 Mutation Test/2013 (Roche)	RT-PCR

Non-small cell lung cancer	Xalkori (Pfizer, NY, USA)	Lysis ALK Break Apart FISH Probe Kit/2013 (Abbott)	FISH

Colorectal Cancer	Vectibix (Amgen, CA, USA)	Therascreen KRAS RGQ PCR Kit/2011 (Qiagen, Venlo, The Netherlands)	PCR
		The cobas KRAS Mutation Test/2013 (Roche)	RT-PCR
		EGFR PharmDx Kit/2003 (Dako)	qlHC

Cystic fibrosis	Kalydeco (Vertex, MA, USA)	Unbranded laboratory test used during (1989)	Clinical trials

Human immunodeficiency virus (HIV)	Ziagen (Viiv Healthcare, Brentford, UK)	LDTs at time of development (2007)	

Human immunodeficiency virus (HIV)	Selzentry (Viiv Healthcare)	Trofile Test/2008 (Labcorp, NC, USA)	qPCR

*Selection of data was based on availability of data, robust prior research, large clinical database, and US market approval.*

*SemiqIHC, semiquantitative immunohistochemistry; CISH, chromogenic in situ hybridization; FISH, fluorescence in situ hybridization; qFISH, quantitative fluorescence in situ hybridization; RT-PCR, real-time polymerase chain reaction; qPCR, quantitative polymerase chain reaction.*

A list of feasible factors impacting the companion diagnostic industry available for systematic assessment was determined from a preliminary overview of existing research (Trusheim et al., 2011) for each of the seven personalized therapeutics in **Table 1**.

Nine factors were analyzed that could influence companion diagnostic development and adoption: CDx price, CDx sensitivity, price of drug standardized per course of treatment, duration of drug treatment, years on the market for drug (measured from year of FDA approval to year of patent expiration, or predicted patent expiration for drugs most likely to receive extended protection), drug efficacy, total current population of patients in specific indication, subpopulation of patient population (calculated with the percentage of total patient population predicted to have the specific mutation or biomarker expression), and response rate of patient subpopulation to drug after selection with companion diagnostic.

Factors impacting both the companion diagnostic manufacturers (price of CDx, sensitivity, total patient population) and pharmaceutical therapy development and adoption (drug prices, effectiveness, patient subpopulation, response rate) were included as factors for CDx market analysis because of the unique regulatory dependency of CDx and personalized therapeutic. The factor “price of drug” was standardized as the total cost for one course of complete treatment. “Drug efficacy” was based on the percentage reported in clinical trials of the drug to either decrease tumor size or reach a standard endpoint such cell count and forced expiratory volume in 1 s. Factors fell into two categories – those relating to the CDx (CDx price, CDx sensitivity) solely and those relating to the therapeutic and treatment (drug price, duration of treatment, years the drug has been on market, drug effectiveness, total patient population, treatable subpopulation, response rate).

### Data Collection and Statistical Analysis

Data in each category for each therapy was individually obtained from eighty distinct sources, ranging from literature searches in PubMed, personal correspondence with diagnostic and pharmaceutical company representatives, current articles in prominent news journals, government dockets, annual reports, patent filings, Internet searches, and published regulatory agency documents, to official FDA released drug labels. Data for factors in the therapeutic market was applied to each CDx. For example, the numerical value for total population of patients with breast cancer was identical for each of Herceptin’s six FDA approved CDx.

Statistical analysis was conducted using JMP. The relationship between two factors was determined by Spearman’s Rho, a non-parametric tool to determine correlation for data without a Gaussian distribution. Significant relationships were determined by *t*-test on regression, with a null hypothesis that the regression is zero. Results were considered significant if *p* < 0.05. Relationships for all possible pairs of factors were tested in order to preserve an unbiased analysis. Statistical analysis was supplemented with detailed case studies examining competition within the CDx landscape in the two CDx/Rx pairs with multiple FDA approved CDx at the time of this assessment (Herceptin with six CDx and Vectibix with three CDx).

## Results

Statistical analysis for 36 hypotheses resulted in two significant relationships and 34 non-significant relationships. **Table 2** shows the nine by nine matrix of factor pairs with calculated *r*- and *p*-values. Further analysis focuses on possible interpretations of insignificant and significant relationships for companion diagnostics, with less focus on relationships for the therapeutic market.

**Table 2:** Correlation (*r*) and significance (*p*) values for financial and product factors affecting the market for companion diagnostic/therapeutic (CDx/Rx) pairs.

	CDx price	CDx sensitivity	Drug price (treatment)	Duration of treatment	Drug years on market	Drug effective-ness	Total patient population	Treatable subpopulation	Response rate
	
**CDx price**	1	**-0.78**	0.18	0.31	-0.54	-0.26	0.25	-0.25	0.07
	0	**0.04**	0.7	0.5	0.22	0.57	0.59	0.59	0.88
		
**CDx sensitivity**		1	0.18	-0.44	-0.04	-0.32	0.2	-0.14	-0.14
		0	0.7	0.33	0.94	0.48	0.67	0.76	0.76
			
**Drug price (treatment)**			1	0.55	0.51	-0.71	-0.31	-0.36	0.46
			0	0.2	0.25	0.07	0.5	0.43	0.29
				
**Duration of treatment**				1	-0.49	-0.29	-0.13	-0.04	0.45
				0	0.27	0.53	0.78	0.94	0.31
					
**Drug years on market**					1	0.29	-0.3	-0.21	0.11
					0	0.53	0.51	0.66	0.81
						
**Drug effectiveness**						1	0.41	0.29	0.18
						0	0.36	0.53	0.7
							
**Total patient population**							1	**0.88**	0.11
							0	**0.01**	0.82
								
**Treatable subpopulation**								1	0.29
								0	0.53
									
**Response rate**									1
									0

*Thirty-six factors were statistically analyzed using Spearman’s rank test on JMP. Relationships were significant if p < 0.05. Significant relationships are bolded and underlined. Gray box is correlation while white box is significance.*

### Relationships Affecting Companion Diagnostics

**FIGURE 3:**
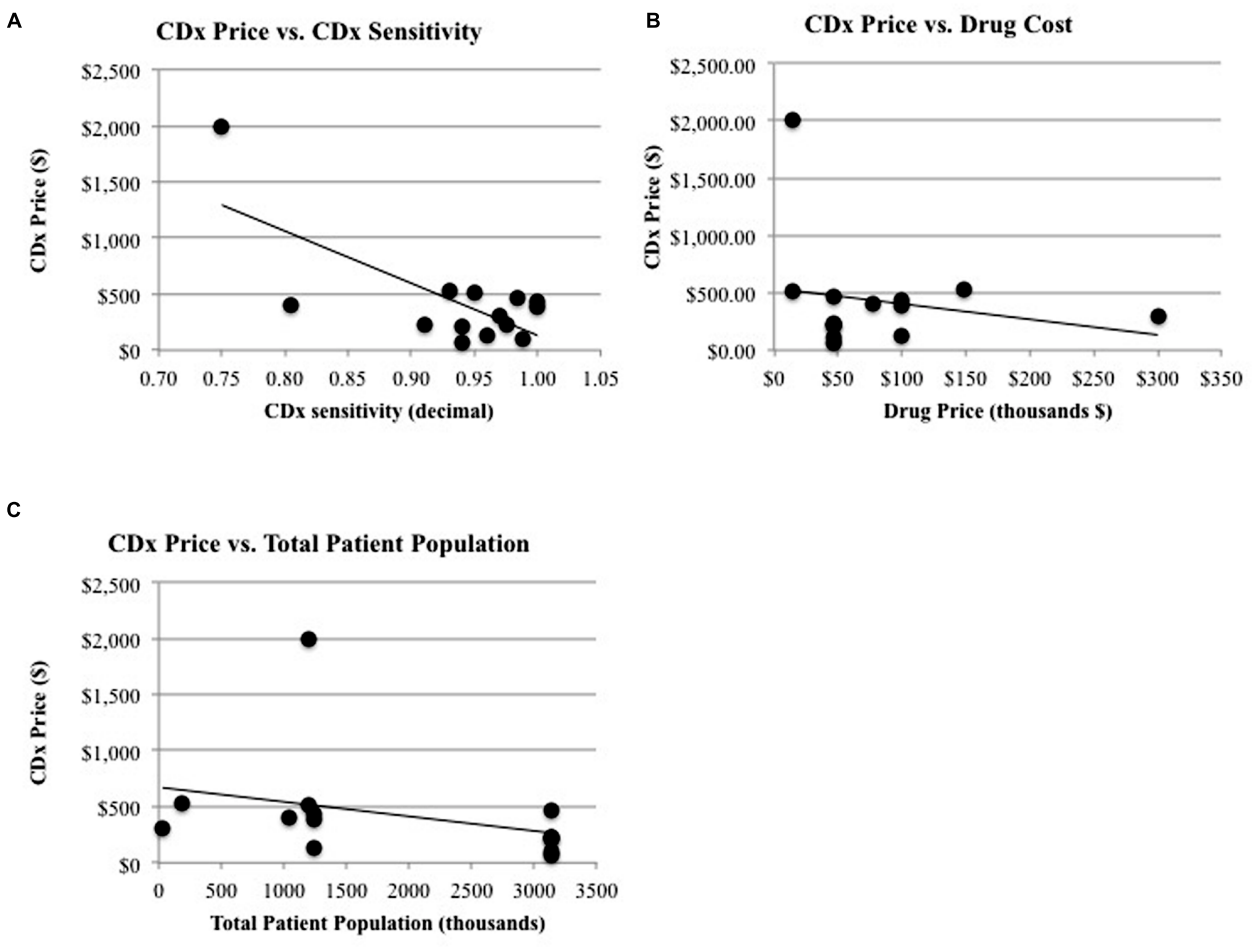
**Significant relationships and non-significant relationships for CDx price. (A)** There is a significant relationship between CDx price and the duration of drug treatment (*R*^2^ = 0.347, *p* = 0.026) and a significant relationship **(B)** between CDx price and CDx sensitivity (*R*^2^ = 0.507, *p* = 0.004). **(C)** There is a non-significant relationship between CDx price and revenue of drug (*R*^2^ = 0.171, *p* = 0.140) and the total patient population (*R*^2^ = 0.105, *p* = 0.256).

The factors that relate to the companion diagnostic have a significant relationship between themselves: the CDx price has a significant relationship with the CDx sensitivity. **Figure 3A** shows this relationship graphically, with rho calculated as a Spearman’s rank correlation that standardizes outliers as opposed to a Pearson’s correlation for Gaussian distributions (*r* = -0.78, *p* = 0.04). One explanation for the price of CDx increasing with increasing sensitivity is that a CDx which can more accurately stratify the patient population into correct respondent groups might take longer to develop, or utilize more expensive technology, and therefore be priced higher to offset the higher costs of development.

The factors that relate solely to the companion diagnostic (CDx price, CDx sensitivity) do not have significant relationships to factors relating to the therapeutic or treatment (drug price for treatment, years on market for drug, drug efficacy, population measurements, and response rates to drug). One may expect a significant relationship to exist between the factors affecting companion diagnostics and the therapeutic or treatment because of their interconnected nature and regulatory co-development. For instance, it may be expected that companion diagnostics would follow the same pricing curve as their corresponding therapeutic, because there are costs involved in meeting regulatory standards (Quinn, 2010). A high risk, high benefit therapeutic such as Zelboraf will be priced to reflect the time, cost, and production of larger clinical trials, more advanced technology, and longer development time and because the companion diagnostic used during clinical trials for Zelboraf faces a similar regulatory pathway, it might be expected to be priced accordingly. However, there is no significant relationship between CDx price and the drug price for a complete course of treatment (*r* = 0.18, *p* = 0.7, **Figure 3B**). Zelboraf costs approximately $56,400 per treatment (Cohen and Felix, 2014) with a CDx priced at $401, while Vectibix costs $68,000 per treatment (Pollack, 2006) – more than Zelboraf – but with a CDx priced lower than $401, at $385 (Ray, 2013). In another instance, it may be expected that CDx price will have a relationship with the treatable subpopulation because the treatable population is a representation of the full market size that a CDx could penetrate. However, market size, measured by the total patient population, does not statistically have a significant relationship with the price of a CDx (*r* = 0.25, *p* = 0.59, **Figure 3C**). The lack of significant relationships raises more research questions and limitations are further discussed in Section “Limitations.”

### Competition

**FIGURE 4:**
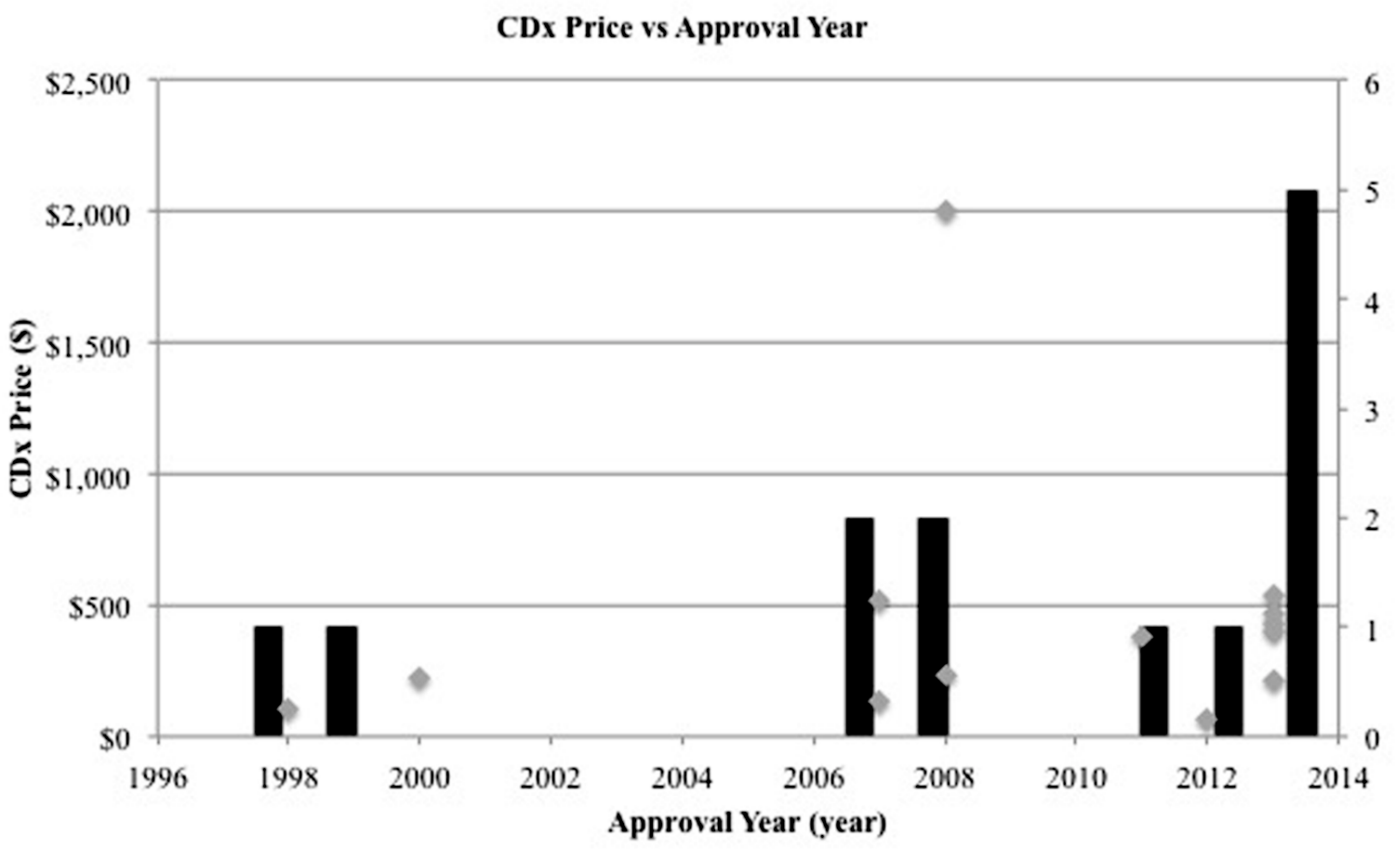
**Number of companion diagnostics approved per year increases.** Gray scatterplot shows the individual year of approval for each CDx in this study. Black bar histogram shows the total number of companion diagnostics approved per year in this study.

**FIGURE 5:**
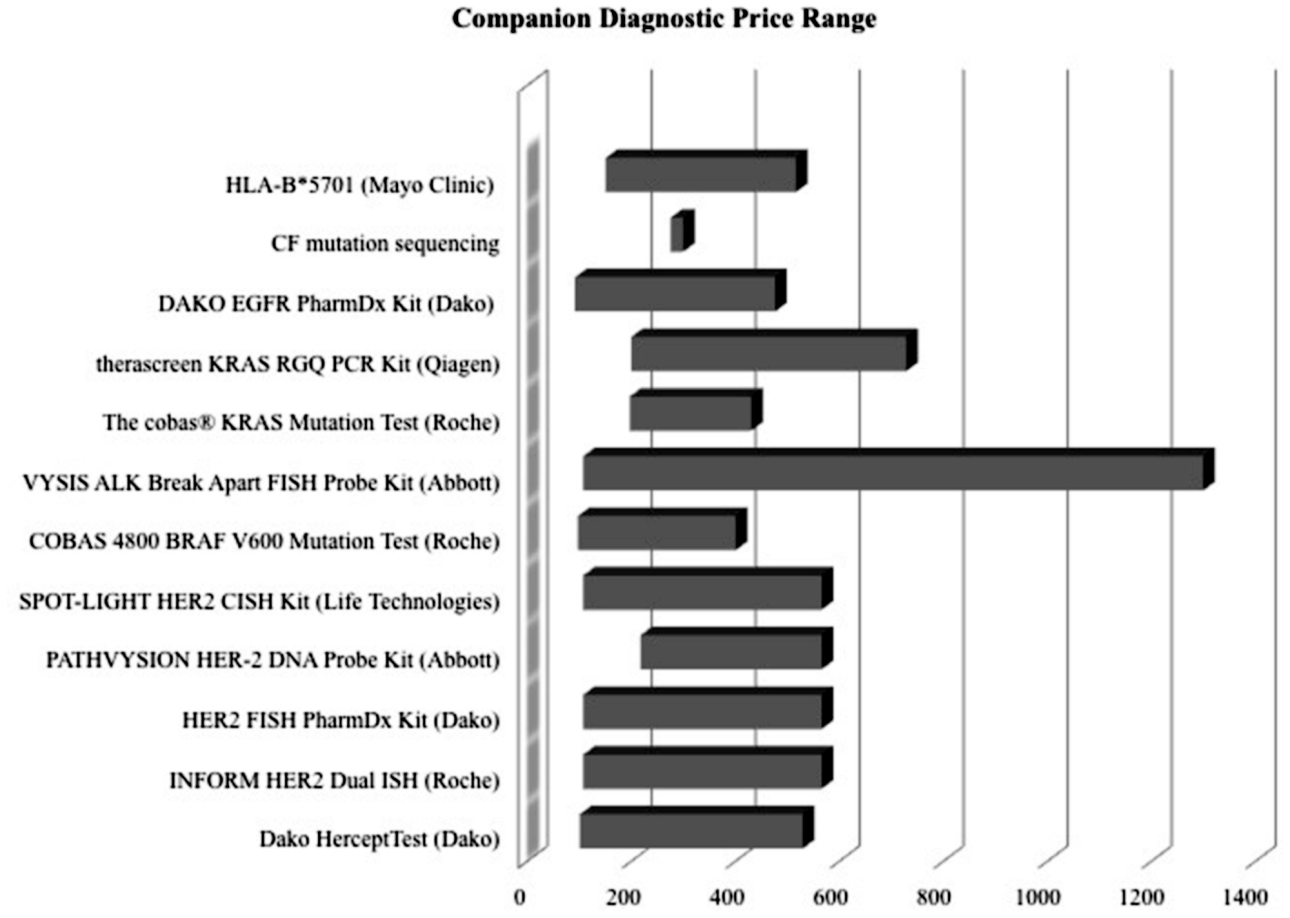
**Price ranges of companion diagnostics are wide.** Lower boundary determined by Medicare reimbursement for the CPT code most closely associated with test. Upper boundary determined typically by list price of company, obtained from a multiple of sources, such as published literature and personal communication.

**FIGURE 6:**
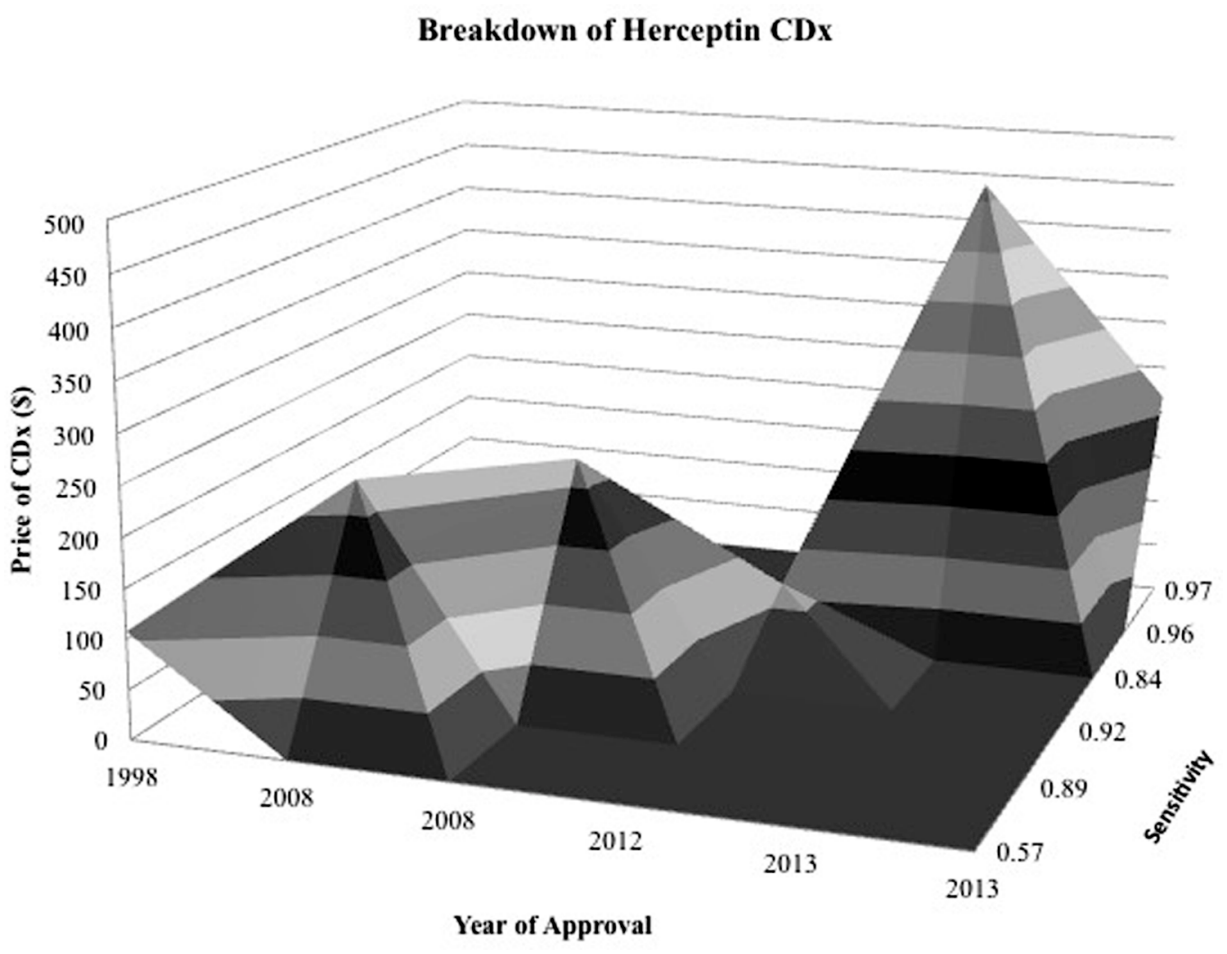
**Surface map for Herceptin CDx shows CDx have an expanding range of prices and greater sensitivity as time goes on.** Z axis is specificity of diagnostic and contour represents the price of the diagnostic in relation to its specificity during the year that it was approved. The more specific and expensive, the further back and elevated the peak.

Examining competition in two case studies of Herceptin and Vectibix revealed that as the number of CDx for one therapeutic increases, sensitivity improves. Since the approval of the first companion diagnostic, there have been a growing number of companion diagnostics each year, with an expanding range of pricing and more diverse test types (**Figures 4** and **5**). In the case study of Herceptin, the price range of companion diagnostics heightened with the expansion of testing methods, and accordingly, sensitivity increased as technology improved through the years (**Figure 6**).

In the case study of Vectibix, the companion diagnostic followed a unique developmental pathway that ultimately led to better identification of the patient population that responded positively to anti-EGFR therapy. Originally, overexpression of EGFR was discovered as a biomarker for colorectal cancer and enlisted in clinical trials for an anti-EGFR mAb, Erbitux. Following the model of Herceptin, patients enrolled in Erbitux clinical studies had to demonstrate immunohistochemistry (IHC) evidence of positive EGFR expression, leading to the co-development of Dako’s EGFR pharmDx^TM^ test [Genentech: Herceptin^®^, (trastuzumab) Development Timeline, n.d.].

In 2007, following the model set by Herceptin and HER2, Erbitux and its companion diagnostic were co-approved. However, unlike the smooth success of Herceptin, subsequent studies post-drug approval failed to support the correlation between EGFR overexpression and response to either Erbitux, or a second anti-EGFR drug in development, Vectibix (Cunningham et al., 2004; Saltz et al., 2004). In a parallel set of studies, a better predictor of response to Erbitux and Vectibix started emerging. Pivotal studies conducted on the clinical utility of a *KRAS* mutation status confirmed the *KRAS* gene as an accurate predictor of non-responsive individuals to anti-EGFR therapy (Lievre et al., 2006). The FDA acknowledged the evidence base for KRAS presence as a marker for patients who would achieve no benefit from anti-EGFR therapy and issued a label update for the entire class of drugs, recommending KRAS testing as part of colorectal cancer patient management (Amado et al., 2008).

Subsequent approval of Vectibix and corresponding companion diagnostic, Qiagen’s (Venlo, The Netherlands) therascreen KRAS RGQ PCR kit, followed (Ray, 2014). Two years later, Roche developed the cobas^®^, KRAS Mutation kit; a companion diagnostic comparable to Qiagen’s therascreen, but one that produces faster results, but at a higher price (Gonzalez de Castro et al., 2012).

## Discussion

Quantitatively assessing the relationship between factors affecting the market for companion diagnostics and corresponding therapeutics reveals that the regulatory codependence of CDx/Rx pairs is not reflected in their economic landscape. With CDx/Rx development, the two separate industries (diagnostic and pharmaceutical) form a relationship in which FDA approval, reimbursement, and prescription of the therapeutic is dependent on accurate stratification of the patient population, and the regulatory approval and utility of the companion diagnostic relies on approval of the therapeutic. Data shows that factors pertaining to the therapeutic do not likewise influence the price or sensitivity of the CDx. It appears that the companion diagnostics market is governed by a different set of drivers than the pharmaceutical market because there is no significant relationship between economic factors for the two industries.

The economic independence of CDx and therapeutic may disincentivize development of companion diagnostics. Personalized medicine is inherently a disruptive innovation and incentives to develop more personalized therapeutics may be economic, as seen through high prices of personalized medicines in oncology. However, the push toward greater investment in CDx seems to be solely regulatory, because CDx do not share in the same high prices and multiple doses as their expensive corresponding therapeutics experience. Pricing for CDx would indicate that diagnostic tests are valued as utility, much like a needle that is used to inject a serum, and not as an integral part of the therapeutic itself (Taube et al., 2009). However, unlike the needle, which is universal and non-specific, the CDx can direct a physician toward a specific course of treatment and is as important to a treatment as the drug itself. While companion diagnostics are clinical tools that can help physicians decide the course of patient treatment, their pricing practices are independent of the pricing practices for their corresponding therapeutic, and this economic discrepancy might be a currently overlooked barrier in the race to develop more personalized therapeutics.

In context, there are also many other barriers to the development and adoption of companion diagnostics. Reimbursement uncertainty continues to create challenges (Buchanan et al., 2013). Current reimbursement for companion diagnostics relies on a complex system of CPT code stacking, private health plan policies, evidence of clinical utility, and cost of both the diagnostic and the treatment (Faulkner, 2009; Trosman et al., 2011). Having a wide range for the ‘price’ of a companion diagnostic indicates a lack of standardization in the charges different providers and insurers receive (**Figure 5**) and could create an unwillingness to adopt complex therapies that require not one, but two different payments – one for the initial companion diagnostic, and one for the following drug treatment.

Coverage and reimbursement for high-risk, high-reward personalized treatments are often decided on a health plan level and the existence of 100s of different insurance plans along with a national payer system for low-income, disabled, and elderly Americans leads to fragmentation in access to life-saving therapies (Redwood, 2002). Skepticism of the efficacy of companion diagnostics to predict responses to therapy and uncertainty on the necessity of a companion diagnostic slows reimbursement and creates challenges to the widespread implementation of existing personalized therapies (Faulkner et al., 2012).

Additionally, the dominance of late stage competition and lack of early stage competition within companion diagnostics creates barriers to the innovation and development of the companion diagnostics market (Amir-Aslani and Mangematin, 2010). Uncertainties in evaluating the utility of an assay when clinical activity of the therapeutic or the relationship of the biomarker to the mechanism of action of the therapeutic being developed are major scientific challenges for multiple companion diagnostics to be developed at once (Taube et al., 2009). The high risk nature of CDx development incentivizes publicly listed diagnostic companies to gravitate toward close-to-market products capable of generating revenues in the short term to satisfy the investment community instead of encouraging early stage technologies that might deliver products far in the future (Dickman, 2013).

The large upfront investment with low certainty in returns for diagnostics does not encourage competition to occur between companion diagnostics for a singular therapeutic before an initial CDx reaches market. Competition can act as a positive force to stabilize prices, and increase innovation (Batchelder and Miller, 2006), and while companion diagnostics market does not currently offer a viable space for competition, it has the potential to.

The predictive power of biomarkers varies between indications and therapies (Taube et al., 2009), possibly opening up space for more biomarkers with stronger analytical power to enter into consideration for a therapeutics’ clinical trials. The presence of some biomarkers, such as a particular gene mutation in a CF mutation panel, or the detection of a KRAS mutation status, allows for straightforward stratification of patients into dichotomous populations – a group that will respond to a therapeutic and one that will not (Cheng et al., 2012). The presence of other genes, such as HER2 or ALK (anaplastic lymphoma kinase), is measured on a more continuous scale and requires multiple runs and expert interpretation before subsequent delineation into one particular treatment therapy (Rogowski et al., 2010).

As seen in the case of Vectibix, for which clinicians began with an EGFR-positive companion diagnostic, then added a KRAS-negative companion diagnostic, a single test may not be sufficient to correctly identify patient response levels. Well-planned incremental evaluations of CDx/Tx pairs across phase I, II, and III studies would ideally increase the efficiency of the drug development process and response to therapeutic (Taube et al., 2009; Jørgensen, 2013).

The inherent complexity of biomarker development and the even more uncertain nature of drug development, innovation models that encourage open competition during early stages might work to increase success rates of therapeutics through clinical trials and lower attrition values (Dickson and Gagnon, 2004). With the structure of co-development, competing development of diagnostic methods is currently limited to established contracts and investments from pharmaceutical companies, but advances in technology and risk sharing models might propel a more independent model for competition in the future.

### Limitations

This study has a number of limitations (**Table 3**). The main limitation of this study is the maturity of the market and therefore the small amount of robust, publically available data published for each companion diagnostic and therapeutic. A young industry has a large number of variations that make direct comparisons difficult. For instance, the point of stratification for one personalized therapeutic is not necessarily the same as the point of stratification of another personalized therapeutic. Some companion diagnostics (Herceptin, Zelboraf, Xalkori) followed a co-development model and received simultaneous CDx/Rx approval from the FDA.

**Table 3:** Abbreviated list of limitations.

Limitations
• Recently approved CDx/Rx pairs • Small amount of robust, publically available data • Young market has a large number of variations ∘ Different entrance points for CDx (co-developed, individually adopted) ∘ Different classification as tests (FDA approved vs. LDT) • Wide range of pricing for CDx • Different endpoints for efficacy measures of therapeutics in different indications • Rapidly changing regulatory and policy landscape • Quickly developing technological advances

*Select limitations are detailed in limitations section.*

Other diagnostics (Vectibix, Ziagen, Selzentry, Kalydeco) were classified as laboratory-developed tests (LDTs) at their conception and received FDA approval after drug approval, or were adopted into clinical practice without FDA review (Kahn et al., 2002; Robertson et al., 2002). Given that LDTs undergo a less rigorous clinical review through the Clinical Laboratory Improvement Amendment (CLIA) regulatory pathway, they may have lower cost requirements, leading to acute cost differences (McCormack et al., 2014).

There are also two factors, CDx price and drug efficacy, with notable caveats and limitations. The factor ‘CDx price’ has many contributing sources. There are multiple types of price determinant for companion diagnostics, with a wide range of pricing (**Figure 6**). The lower boundary of the pricing range is attributed to Medicare National Limitation Amounts published in the 2014 Clinical Laboratory Fee Schedule (Medicare, 2015) while the upper boundary is generally attributable to company list prices and hospital billings for outpatient, non-insured individuals. Price variation also stems from the segmented nature of Medicare reimbursement; while Medicare is a major payer of high-risk, high-benefit therapies, reimbursement differs depending on region and individual contracts.

Further fragmentation occurs on a providers level with various hospitals stacking CPT codes in a multitude of ways (Faulkner et al., 2012). Other forms of price determination within the ranges in **Figure 6** include private insurance, hospital billings, and quotes from individual diagnostic companies. Private insurance often takes example from Medicare limits, and each has individual coverage policies that conditionally cover treatments based on specific situations. Hospitals have opaque pricing policies and differ within regions. Companies typically sell in bulk, rendering prices for individual companion diagnostic tests difficult to determine.

Due to these limitations, no one measure of price is appropriate to represent the complexities in CDx price. Therefore, for the purposes of this analysis, the price of the CDx is defined as the price most associated with the specific companion diagnostic in published, pre-existing literature, and is usually obtained as a measure of the cost of testing equipment and compensation for labor and interpretation.

Therapeutic ‘efficacy’ is defined in the context of medical interventions as “the performance of an intervention under ideal and controlled circumstances” (Singal et al., 2014). Requirements for an efficacy trial include obtaining a readily available form of the drug, recommending the drug to the identified target population, and adhering to the drug prescription. The selected personalized therapeutics in **Table 1** address different indications and have different endpoints – oncology generally has endpoints of tumor reduction or disease free progression, cystic fibrosis has an endpoint of FEV (forced expiratory volume), and HIV has endpoints of response rate or percentage of HIV infected RNA. It is hard to find equivalent measures of endpoints and clinical utility across different indications due to the varied nature of disease and mechanisms of action of therapeutics. Despite the different measures of clinical endpoints, the clinical trials for the personalized therapeutics meets the requirements for efficacy trials with equal access, targeted populations, and high adherence rates. Therefore, data for drug efficacy was appropriated from published clinical trial information on patents, labels, and official FDA recommendations.

Additionally, unclear standards for clinical utility, rapid advances in technology, uncertainty in relative efficiency and cost-effectiveness of CDx, and differences inherent to different indications create a variegated landscape for companion diagnostics (Taube et al., 2009; Frueh, 2010; Davies et al., 2014; Meadows et al., 2015).Given the rapidly evolving regulations involved in the development of companion diagnostics and the number of contracts, investments, and new therapeutics in the drug development pipeline, this assessment should be considered a snapshot of a quickly evolving system. There are many complexities in the technological development and adoption of companion diagnostics, and further research will be needed as the landscape evolves.

## Conclusion

The market for companion diagnostic development and adoption is complex and difficult to navigate. Where there is regulatory pressure to co-develop companion diagnostics for the approval of personalized therapeutics, there is a lack of parallel economic collaboration to develop companion diagnostics. Factors that pertain to the therapeutics market, such as market size and response rate do not drive the CDx market. The discrepancy between the factors for the therapeutic/treatment and the factors for companion diagnostics despite similar regulatory standards, along with an uncertain reimbursement structure and limited opportunities for early stage competition shows that there is a misalignment in economic incentives that could potentially discourage the development companion diagnostics, for unspecified therapeutics. Further research will be needed as the landscape for companion diagnostics evolves.

## Author Contributions

All authors were responsible for conception and design of the experiment, revised the work critically for important intellectual content, approved the final manuscript and agree to be accountable for all aspects of the work. DL and JS collected and analyzed the data and wrote the manuscript.

## Conflict of Interest Statement

The content outlined herein represents the individual opinions of the authors and may not necessarily represent the viewpoints of their employers. David A. Brindley is a stockholder in Translation Ventures Ltd. (Charlbury, Oxfordshire, UK) and IP Asset Ventures Ltd. (Oxford, Oxfordshire, UK), companies that, amongst other services, provide cell therapy biomanufacturing, regulatory and financial advice to pharmaceutical clients. James A. Smith is a consultant of IP Asset Ventures Ltd. David A. Brindley is subject to the CFA Institute’s Codes, Standards and Guidelines, and as such, must stress that this chapter is provided for academic interest only and must not be construed in any way as an investment recommendation. Additionally, at time of publication, David A. Brindley and the organizations with which he is affiliated, may or may not have agreed and/or pending funding commitments from the organizations named herein.
