# Corrigendum: A Quantitative Assessment of Factors Affecting the Technological Development and Adoption of Companion Diagnostics

**DOI:** 10.3389/fgene.2016.00104

**Published:** 2016-06-07

**Authors:** Dee Luo, James A. Smith, Nick A. Meadows, Anna Schuh, Katie E. Manescu, Kim Bure, Benjamin Davies, Rob Horne, Mike Kope, David L. DiGiusto, David A. Brindley

**Affiliations:** ^1^Department of Biological Basis of Behavior, University of PennsylvaniaPhiladelphia, PA, USA; ^2^The Oxford—University College London Centre for the Advancement of Sustainable Medical Innovation, The University of OxfordOxford, UK; ^3^Nuffield Department of Orthopaedics, Rheumatology, and Musculoskeletal Sciences, University of OxfordOxford, UK; ^4^KinapseLondon, UK; ^5^Oxford National Institute of Health Research, Biomedical Research Centre, Molecular Diagnostic Centre, Oxford University HospitalsOxford, UK; ^6^Department of Biochemical Engineering, University College LondonLondon, UK; ^7^Nuffield Department of Orthopaedics, Rheumatology, and Musculoskeletal Sciences, Botnar Research Centre, University of OxfordOxford, UK; ^8^Sartorius StedimGöttingen, Germany; ^9^The UCL School of Pharmacy, University College LondonLondon, UK; ^10^SENS Research FoundationMountainview, CA, USA; ^11^Stem Cell and Cellular Therapeutics Operations at Stanford University Hospital and ClinicCalifornia, CA, USA; ^12^USCF-Stanford Center of Excellence in Regulatory Science and InnovationCalifornia, CA, USA; ^13^Centre for Behavioural Medicine, UCL School of Pharmacy, University College LondonLondon, UK; ^14^Harvard Stem Cell InstituteCambridge, MA, USA

**Keywords:** companion diagnostic, combinational therapy, risk:benefit appraisal, healthcare risk management, personalized medicine, stratified medicine, healthcare translation, commercialization

With regards to Figure 3: Significant relationships and non-significant relationships for CDx price, the graph (A) of CDx Price vs. CDx Sensitivity as well as the corresponding legend, is in error. Graph A wrongly depicts a trendline unadjusted for outlier effect, and the correct graph, as described in the text, is shown below. The corresponding legend has been likewise corrected to reflect the correct graph title and statistical values described in the text. This correction does not affect the scientific validity of the results, as the discrepancy was with the presentation of results.

**Figure 3:**
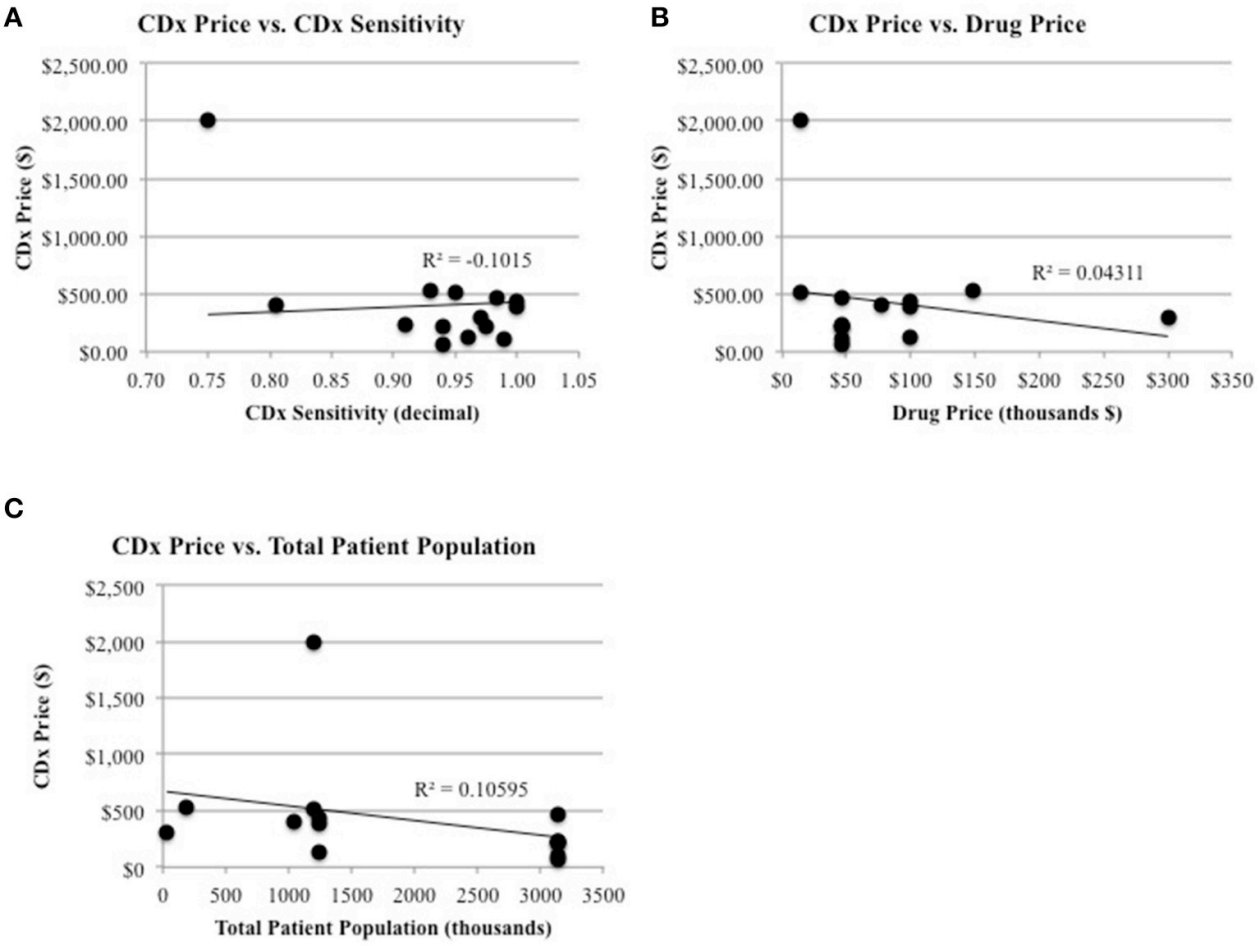
**Significant relationships and non-significant relationships for CDx price. (A)** There is a significant relationship between CDx price and CDx sensitivity (*R*^2^ = −0.10, *p* = 0.04). **(B)** There are non-significant relationships between CDx price and drug price (*R*^2^ = 0.043, *p* = 0.70) and **(C)** the total patient population (*R*^2^ = 0.105 *p* = 0.59).

## Author contributions

All authors listed, have made substantial, direct and intellectual contribution to the work, and approved it for publication.

### Conflict of interest statement

The authors declare that the research was conducted in the absence of any commercial or financial relationships that could be construed as a potential conflict of interest.

